# Muscle Tone, Stiffness, and Elasticity in Elite Female Cyclists after Consecutive Short Competitions

**DOI:** 10.1155/2024/2434494

**Published:** 2024-04-02

**Authors:** Cristina Rotllan, Francisco Corbi, Ginés Viscor

**Affiliations:** ^1^Secció de Fisiologia, Departament de Biologia Cel·lular, Fisiologia i Immunologia, Facultat de Biologia, Universitat de Barcelona, Barcelona, Spain; ^2^Institut Nacional d'Educació Física de Catalunya, Universitat de Lleida, Lleida, Spain

## Abstract

**Background:**

For professional road cyclists, most overload injuries affect the lower limbs. They are mostly represented by contractures or muscle shortening, characterised by a variation of muscular tone, stiffness, and elasticity. This real-life study aimed to assess specific mechanical parameters in top-class female cyclists who participated in 3 races a week. *Hypothesis*. Muscle tone, stiffness, and elasticity will be affected immediately after competition and at the end of the week due to accumulated fatigue.

**Methods:**

Six professional cyclists were evaluated. This pilot study consisted of a controlled trial and three days of competition, with rest days between them. MyotonPRO was used to measure tone, stiffness, and elasticity in six leg muscles: vastus lateralis (VL), vastus medialis (VM), rectus femoris (RF), biceps femoris (BF), lateral gastrocnemius (LG), and medial gastrocnemius (MG). Daily basal and pre- and postrace measures were carried through to the 3 races in a week.

**Results:**

The muscular tone of VL, VM, LG, and MG and the stiffness of VL, VM, RF, BF, LG, and MG decreased after races. VL and RF were mostly affected by (*p*=0.05) and (*p*=0.009), respectively. Basal elasticity improved over time until the last day.

**Conclusions:**

Muscle tone and stiffness decreased after a very intense and exhausting cycling endurance competition. Basal elasticity improved immediately after the race and continued this trend until the end of the week. More research is needed on changes in mechanical properties in competition and risk prevention of injuries.

## 1. Introduction

Several studies have examined the most common injuries in amateurs [1–4], where the incidence of overtraining injuries can reach 85% [4]. However, only four epidemiological studies, all retrospective, have focused on studying male professional cyclists [5–8]. Regrettably, the definition of injury, severity, and specificity of the diagnosis were different among them.

However, no epidemiological study has observed how competition affects professional female cyclists. Female representation in research in exercise and sports medicine is limited; only 39% of the participants in studies published for 3 years in relevant journals were women [9]. In addition, gender-dependent differences in the probability of suffering certain injuries have been found; for that reason, it is necessary to include more women in the study samples [4].

Professional cyclists have a high risk of traumatic and overuse injuries (0.5 per year/cyclist), considering both traumatic and overuse injuries [6]. This means that at least one in two cyclists is exposed to an injury each season, which is considered a high risk of injury.

Regarding overuse injuries, patellar and achilles tendinitis seem to be the most common injuries. In addition, the rate of overuse injuries affecting the knee was 62% of all overuse injuries [5].

Later, a study exclusively describing overuse injuries in 109 cyclists from 7 professional teams throughout a season reported how lumbar and knee pain were the most commonly reported injuries, with 58% and 36%, respectively. Unfortunately, no specific clinical diagnosis was established [7], and various injuries, such as muscle contractions and shortening, are only considered when they limit the ability to train and compete [10].

De Bernardo and colleagues observed how, of the 112 injuries suffered by 51 professional road cyclists over 4 years, 51.5% were overtraining and 26.4% were overuse injuries located in the muscles, causing these chronic muscle contractions and shortenings and mainly affecting the gastrocnemius and quadriceps [6].

Contractures are the most common injuries between muscle overload injuries and are persistent, painful, and involuntary contractions of one or more muscles, which represent a defensive reaction when the muscle is under stress [11]. From a microscopic point of view, when a muscle is under contraction, sliding of the actin and myosin filaments can be seen; however, this is not preceded by activation of the neuromuscular junction. For this reason, contractures can sometimes also be called in other ways, and it is considered that they may have a multifactorial cause [12]. For example, an intracellular Ca^2+^ build-up will cause more active cross-bridges, as well as the action of chemical agents such as high doses of caffeine [12].

Many international road cycling competitions consist of consecutive daily races, meaning it requires a quick and satisfactory recovery between days to reach maximum performance during competition [13].

On the other hand, there is a relationship between overuse injuries, fatigue, and altered muscle tone. People with contractures feel an involuntary increase in muscle tone and a decrease in elasticity, which seems to limit their range of motion [11, 14]. Muscle tone is commonly defined as tension at rest, clinically determined as resistance to passive movement [15], and can be evaluated, in superficial muscles, by tensiomiography [16, 17] and myotonometry tools [18] MyotonPRO® is an easy-to-use, lightweight, noninvasive device outside the laboratory employed in this study. Another method such as tensiomyography also assesses muscle properties noninvasively.

These instruments allow to record viscoelastic properties of muscle tissue that are not constant but depend on previous movements, where the viscoelastic tone is a typical expression of the thixotropic nature of muscles and myofascia [19]. The relationship between muscle contractures and injuries has been observed in different studies, along with increased muscle stiffness, which can cause the onset of more serious damage and pain, particularly after intense training [20] or after international consecutive daily races.

Furthermore, many studies have attempted to assess the mechanical response of the muscle, the risk of injury in athletes, and how these properties vary depending on physical exercise [21–23]. The relationship that has been observed between muscle tone, elasticity, and muscle stiffness with contractures can help in the early detection of these or even in the prevention of more serious injuries that involve functional incapacity or stoppage of the competition. In this study, we hypothesize that accumulated fatigue during a cycling competition could modify muscle properties. Considering that the muscle groups most affected in cycling are those of the legs and taking also into account that the variation in muscle tone, stiffness, and elasticity could influence a higher incidence of muscle contractures, the main objective of this pilot study was to analyse, in professional cyclist women, how muscle properties are affected by the completion of a 5-day stage competition.

## 2. Materials and Methods

### 2.1. Participants

A group of six professional female cyclists from one of the UCI World Tour teams, the highest international women's category, took part in this study.

The inclusion criteria were to be part of one of the participating teams and have at least one year of cycling experience. The exclusion criteria were to finish the competition with some type of muscle or joint discomfort and have some injury or muscle ailment before the competition.

Anthropometric measurements and years of cycling experience were collected (Table 1). None of the participants took hormonal contraceptives. The study was designed following the standards of the Bioethics Committee of the University of Barcelona and in accordance with the principles of the Declaration of Helsinki of 1975, revised in 2008.

### 2.2. Noninvasive Muscle Properties

MyotonPRO® (Myoton Ltd., Estonia) was used to assess muscle tone, muscle elasticity, and stiffness. MyotonPRO® is a noninvasive, portable, easy-to-use, and relatively inexpensive device, which is used for prevention, detection of the risk of injury, and monitoring in rehabilitation processes. These characteristics allow it to be used in many environments, including in competitions and outside of a laboratory [18, 24].

**Figure 1:**
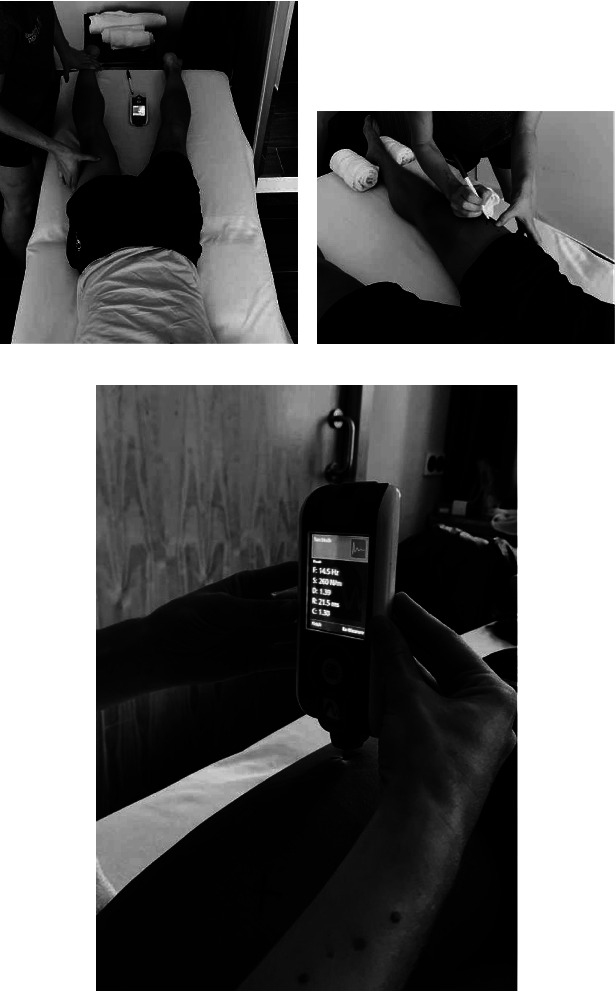
Technique and measurement position of MyotonPRO® exploration. (a) Identification of measurement location by isometric contraction (b) marking the measurement point with a permanent marker (c) measurement point with Myoton PRO®.

The device was configured with a preload of 0.18 N. The device was placed perpendicular to the belly of the muscle to be evaluated, with a precompression of the subcutaneous tissue performed independently of the subject taking the measurements (Figure 1). The device delivered a brief (15 ms) low mechanical impulse (0.4 N) followed by a rapid rest and release of the applied force [21]. This impulse causes an elastic deformation in the muscle tissue that shows oscillations that are recorded and saved by the device.

During the test, the following parameters are obtained simultaneously: (a) frequency of oscillation (Hz) which indicates the muscle tone or intrinsic tension of a muscle, (b) muscle stiffness (N/m) which is the muscle's ability to resist an external force or its contraction, which modifies its shape [25]; logarithmic decrement (log D) which describes the muscle elasticity, namely, the ability to recover its shape after being deformed or after its contraction [24].

### 2.3. Study Design

The participants competed in 3 cycling races in a week: La Vuelta a Navarra (2 stages with a day of rest interspersed between the two stages) and the one-day race Donostiako Klasikoa, with a day of rest between the two competitions. The first race of la Vuelta a Navarra was 108 km in distance and had 2.297 m of elevation gain; the second race was 120,7 km in distance and 1.321 m in elevation gain. And the last, Donostiako Klasikoa race was, 126,7 km in distance and 2.693,3 m elevation gain.

This repeated measure study design consisted of a familiarization phase, pre- and postcompetition measurements and baseline evaluations from the first to the fifth day of the international cycling competition week (Table 2).

The familiarization phase (FP) was carried out in the first competition of the three stages that the cyclists participated in for 5 consecutive days. During the FP, it was possible to assess the intrinsic characteristics of participating in a professional category cyclist race (place and environment where the measurements were taken, the time needed to perform them, and the transfer time from the start point to the finish point of the race) and to observe the possible unexpected events or aspects beyond our control (climate, traffic incidents, or road closures).

During the two subsequent competition days (C1 and C2) measurements were taken, a maximum of 40 minutes, before and after the races (pre- and postcompetition data).

### 2.4. Recording of Muscle Properties

The muscles studied were m. vastus lateralis (VL), m. vastus medialis (VM), m. rectus femoris (RF), m. biceps femoris (BF), m. lateral gastrocnemius (LG), and m. medial gastrocnemius (MG) of the dominant lower limb of the athletes. We identified the dominant leg by asking “If you should shoot the ball on a target, which leg would you use to shoot the ball,” which has been reported to be accurate for symmetrical sports [26].

Figure 1 shows how the samples were taken with cyclists lying on the stretcher in the supine position for the VL, VM, and RF muscles and in the prone position for the BF, LG, and MG muscles. To maintain the posture of the leg in a neutral position, rolled towels were used on each side of the tibial malleolus and on the external part of the limb to be studied in supine decubitus and prone decubitus with the foot out of the stretcher. In the first assessment of the week, the measurement points were marked with a permanent marker. The muscles to be studied had previously been positioned by asking for an isometric contraction, and the evaluation points were selected considering the SENIAM recommendations [27–29]. Furthermore, baseline assessments were performed for 5 days, after getting up and before breakfast.

Table 3 shows the averages and maximum values of heart rate (HR) and watts of power (W) for the 3 cycling races. These values indicate that the subjects performed a high-intensity exercise and that they are elite athletes.

### 2.5. Statistical Analysis

The open-source statistical program JASP 0.10.2 was used for the statistical analysis. A descriptive analysis of the variations in muscle tone, muscle stiffness, and elasticity was carried out. Mean values and standard deviations (SD) were calculated for results that had a normal distribution. For data showing a deviation from normality, the median is considered. The Shapiro–Wilk test was used to observe whether the normality criteria were met (*P* < 0.05). The Student's *t*-test and Wilcoxon test were used to compare pre- and postcompetition situations (C1 and C2) and baselines on day 1 and day 5, in the six muscles studied (VL, VM, RF, BF, LG, and MG). The parameters included were dependent variables (muscle tone, stiffness, and elasticity), and the subject factors were the six muscles of the six cyclists. Large effects of muscle tone and stiffness were found in pre- and postcompetition 1 and 2 and also of elasticity in the basal situation, whereas negligible effect size of muscle tone and stiffness were seen in the basal situation. Effect sizes were provided by calculating the Rang-Biserial Correlation (Rank-Biserial Correlation *r*_*B*_).

## 3. Results

### 3.1. Muscle Properties before and after Competition (C1/C2)

Our data show how tone and stiffness decrease significantly (*p* < 0.001) after the two competitions and muscle elasticity improves once the competitions are over (*p* < 0.001).

**Figure 2:**
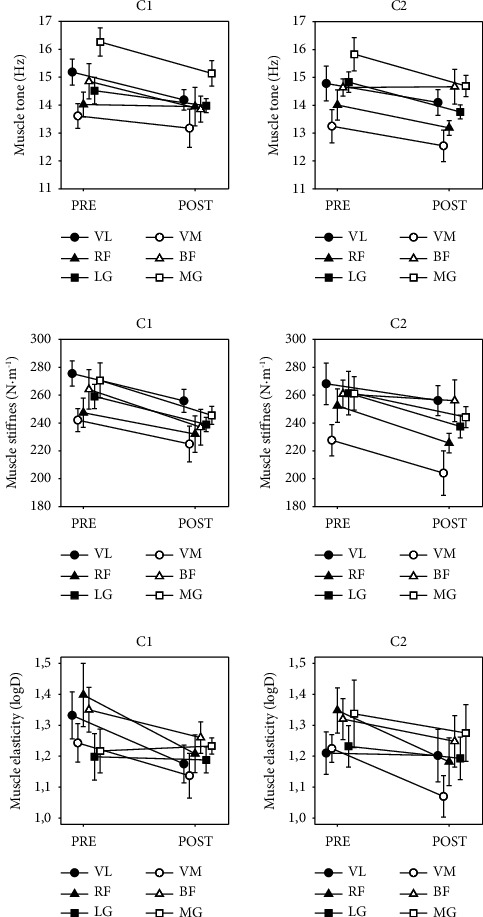
Changes of muscle tone, stiffness, and elasticity before and after competition 1 and competition 2 of 6 muscles.

It is worth noting the tendency, although not always significant, of all muscles to decrease muscle tone postcompetition. In terms of muscle stiffness and a similar evolution of muscle tone, the results showed that all assessed muscles independently decreased muscle stiffness significantly after races. Elasticity tended to increase, decreasing its logarithmic values after competition (Figure 2).

The VL muscle was the only one to undergo significant changes in the three variables to be studied. Its tone was significantly decreased in the postcompetition measurements compared to the precompetition values, in both competitions. Therefore, after C1 dropped from precompetition 15.43 to postcompetition 14.57 (*p*=0.031), as well as after C2 from precompetition values 14.78 ± 1.52 to postcompetition 14.10 ± 1.14 (*p*=0.043). The muscle stiffness of the VL also obtained a significant decrease in C1 (*p*=0.009), from initial values of 275.50 ± 22.13 to final values of 255.90 ± 20.27, and the logarithmic value was reduced in C1, which means that the VL improved its elasticity (*p*=0.023).

RF was the only muscle that improved its initial elasticity after both races, decreasing from the beginning of C1 (1.40 ± 0.25) to a lower value at the end (1.21 ± 0.15) (*p*=0.015) and also dropping from the start of C2 (1.35 ± 0.18) to about 15% (1.18 ± 0.19) after the race (*p*=0.010).

The other muscles MG, LG, BF, and VM also underwent significant modifications to the parameters studied after the competitions, following the same general trend (Table 4).

### 3.2. Muscle Basal Properties

The measurements of the muscle properties were taken throughout the 5 days of competitive concentration, before breakfast, and the values of day 1 were compared with day 5 (Table 5).

The results showed that muscle tone during competition week has individual tendencies concerning the muscles and with various oscillations throughout the week depending on whether it was a competition day or a rest day. However, no significant results were found in any muscle when comparing the values of the first day with those of the fifth.

**Figure 3:**
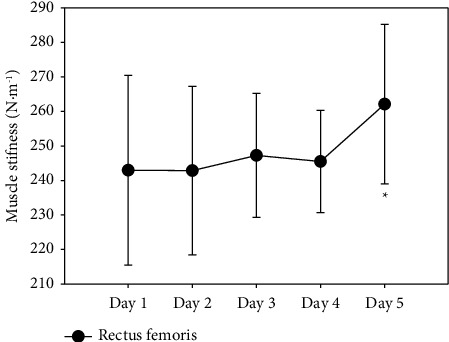
Changes of m. rectus femoris stiffness from day 1 to day 5.

The RF is the only muscle that progressively increased its stiffness throughout the week and ended the last day (262.13 ± 23.11) with a significantly higher value (*p*=0.032) compared to the first day (242.97 ± 27.47) (Figure 3).

Elasticity increased significantly throughout the week (*p*=0.011), regardless of muscle, with a median on day 1 of 1.37 and on day 5 of 1.29.

**Figure 4:**
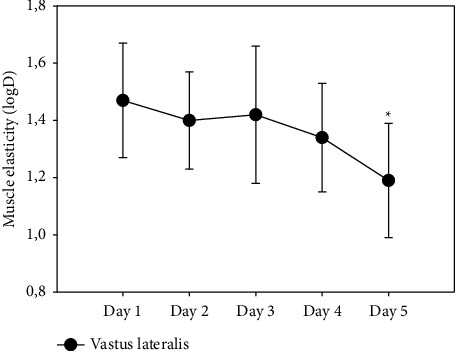
Changes of m. vastus lateralis elasticity from day 1 to day 5.

The VL muscle improved its elasticity (*p*=0.020), decreasing the logarithmic value with a downward trend from the first day (1.47 ± 0.20) to the fifth (1.19 ± 0.20) (Figure 4).

## 4. Discussion

Previous studies used MyotonPRO® as a monitoring tool to prevent injuries and improve performance in sports [22, 23]. However, to our knowledge, there is no other study conducted on professional cyclists that evaluates muscle properties immediately before and after a 5-day race of competition.

The results presented in this study show a decrease in muscle tone, stiffness, and logarithmic decrement immediately after finishing the race compared to levels before the race, which confirms the effect that competition has on the muscles involved in pedalling (Figure 2). Although more studies are needed, it seems like a decrease in tone and stiffness postexercise can be caused by at least 2 different causes: (a) a stress factor before and during competition [30, 31] and (b) postcompetition neuromuscular fatigue [17, 32].

According to several studies, muscle tone is affected by what is called muscle tension [19]. At turn, muscle tension depends on psychological factors, such as stress or anxiety related to an increase in muscle activity. Based on a critical review of the literature on muscle tension and generalized anxiety disorder, there are different proposals. One of them is the excessive concern that can lead to maintaining certain postures and static tension in the muscles for a long time. An example known to many people is the experience of feeling tense muscles after an activity that requires high levels of concentration and cognitive effort, such as an academic exam. To objectify this hypothesis, some researchers propose measuring muscle tension with EMG while subjects watch recordings with worrying or distressing content [33]. Other authors have demonstrated the association between work stress and muscle tension [34, 35]. Chronic myofascial pain, the so-called trigger points responsible for muscle pain, has also been observed to show higher EMG activity during a mental stress situation than in a control one [36].

Several studies reinforce the idea that competitive states lead to stressful conditions, as in the case of our study.

Profuse scientific evidence is available on the relationship between heart rate variability (HRV) and anxiety in precompetition situations [31, 37, 38]. In a recent study, hemodynamic parameters and heart rate variability (HRV) were studied in a group of cyclists. HRV data were collected 5 days before a competition and reassessed before the start of the race, also applying the scale called the “Competitive State Anxiety Inventory-2” (CSAI-2), which is known to be effective in the assessment of anxiety in sports [39]. The results confirmed that cycling competition is a stressful situation capable of altering autonomic nervous control of hemodynamic parameters. Significant changes in HRV are accompanied by alterations in precompetitive anxiety measured before a bicycle motocross (BMX) competition [31], and similar results were found in other disciplines such as swimming [37].

Although stress in a competitive situation is well known, more studies are needed to compare the different competitive moments and how they affect stress, as well as the relationship between stress and muscle mechanical properties.

Another aspect that can affect muscle tone and stiffness after intense exercise is neuromuscular fatigue.

The results of this study demonstrate the decrease in muscle tone and stiffness, in different muscles, after the competition (Table 4), suggesting that the intensity of effort, muscle fatigue, dehydration, changes in skin temperature, and alteration of skin conductivity are some of the possible causes that can justify this behaviour [17].

In many studies, muscle fatigue has been assessed with static monitoring, measuring substrates, metabolites, or other chemicals using biochemical techniques, such as lactate, creatine phosphokinase, hormones, or EMG. Our data agree with previous studies that have used tensiomyography (TMG) to examine the relationship between long-distance running and neuromuscular fatigue, by assessing muscle fatigue after an ultratriathlon in Lanzarote [17]. In this study, significant differences (*p*=0.006) were found in the increase in maximum radial displacement or muscle belly deformation, which represents a loss of muscle stiffness of the biceps femoris muscle, after sport, and the same downward trend of the rectus femoris. These results support our findings, where the stiffness of RF decreased in C2 (*P*=0.009) and BF in C1 (*P*=0.03) (Table 4). It should be noted that in this study, the authors took the precompetition measurements the day before the race, unlike our study, eliminating the possible precompetition stress factor, and hypothetically, the significance would be greater.

Several studies have linked high muscle deformation values with a lack of muscle mass or significant muscle fatigue [40, 41].

In our study, muscle stiffness was reduced in the VL, RF, BF, LG, and MG muscles (Table 4). These results agree with those found by Viitasalo et al. [42], who observed a decrease of 30–35% of the integrated EMG activity of m. vastus lateralis and m. vastus medialis during a maximal isometric contraction, after an 85 km cross-country skiing race, and with St Clair Gibson et al. [43], who examined neuromuscular activity during 100 km of cycling with high-intensity intervals between 1 and 4 km., where power output, EMG measurements, and muscle glycogen were analysed. Unexpectedly, EMG activity decreased in parallel with a reduction in power output, and this phenomenon augmented with time, until the 100 km was completed [43].

In another study with ultramarathon runners, after a 65 km race [44], maximal voluntary contraction (MVC) and neuromuscular fatigue of the knee extensor and plantar flexor muscles were tested, a significant decrease in MVC (*p*=0.005), mainly because the maximum voluntary activation decreased (*p*=0.001) in the knee extensor muscles. The decrease in MVC after activity would match the results of our study. In addition, clear damage to muscle fibres has been observed after long-distance competitions, where the exercise was strenuous [45]. Opposite results were found after strength training, where muscle parameters increased after training [46, 47].

Other factors can also explain how fatigue causes a decrease in muscle mechanical response [32, 48, 49]. Probably, the most important factors are the accumulation of H^+^ and inorganic phosphate, a limitation of the release of Ca^2+^ from the sarcoplasmic reticulum [32], a reduction of the sensitivity of myofibrils to Ca^2+^, and a decrease in the number of cross-bridges [50, 51].

Another possible cause of fatigue is the decrease in neural inputs since it has been observed that, in prolonged exercises, there is a reduction in corticospinal impulses that affect motor neurons [52]. Furthermore, the high oxidation of fatty acids after long-term exercise suggests that free tryptophan in the brain could contribute to this reduced activation [53]. On the one hand, free tryptophan is a precursor to serotonin, and on the other, increased serotonergic activity can induce fatigue by inhibiting the dopaminergic system. Modifications of the oxidative substrate may have led to a decrease in corticospinal impulses [44].

Changes in cortical excitability, such as modification of afferents from the muscle spindle, can reduce the stretch reflex produced by intense cyclic stretching-shortening exercises [54], specifically affecting the intrafusal fibres, and the viscoelastic properties of the muscle. Furthermore, the effects of muscle fatigue on the responsiveness of the Golgi tendon organ and its influence on the alpha motor neuron are not entirely clear [55].

The third parameter observed is elasticity, characterised by the logarithmic decrease in the oscillations performed by MyotonPRO® in the tissue. It describes the dissipation or loss of mechanical energy when the tissue recovers its shape after deformation. The results of our study show that in the VL muscle, the value after the race is lower than before starting and improves elasticity independently of the muscles throughout the week (Figure 4). The lower the logarithmic value, the less energy is needed to modify the tissue, and the more elastic this muscle is [56].

Our study showed significant results of increased elasticity of RF in C1 (*p*=0.015) and C2 (*P*=0.010). The VL increased its elasticity in C1 (*p*=0.023) and the VM in C2 (*p*=0.036) (Table 4).

A previous study showed an almost significant increase in elasticity over 7 days of elite male cycling competition in the RF and BF muscles [21]. The authors justified this tendency as an effect of the plausible expression of tone and stiffness. These results can be understood by the relationship between these two muscles. RF and BF, as two antagonistic muscle groups (BF causes knee flexion and RF extension), are involved in opposite movements around the same joint, which could alter.

A very important factor for an athlete, which participates in his ability, is the recovery index of the muscle working capacity, the relaxation phase [57]. From the point of view of biomechanics, an elastic muscle should recover its initial shape more efficiently. Energy is needed to overcome the resistance to the viscoelastic properties of the antagonist and the contracting muscle [58, 59]. Elastic energy is stored in the muscle during a muscle shortening-stretching cycle to be used in a dynamic movement [60]. One could speculate that the more elastic a muscle is, the less energy is lost in contraction or stretching during the relaxation of the antagonist. Here, we found a tendency for the VL and RF muscles to decrease tone and stiffness after competition, being one of the most activated muscles in cycling [61, 62]. The variability of the competition itself is also a key factor that can affect the muscles differently, depending on whether the stage of the race involves mountain passes or takes place on flat terrain, namely, the standing or sitting position on the bike [63, 64]. Although we did not dive into these aspects in the present study, more research is needed focusing on this issue. We observed how elasticity throughout the week, regardless of muscles, increased significantly (*p*=0.011), and its logarithmic value decreased. Only VL significantly increased its elastic property at the end of the competitive week (*p*=0.020) (Figure 4). These results agree with those found in a previous study; during 7 days of a male cycling competition, the muscles with the best elasticity were BF and RF [21].

Interestingly, the evolution of basal stiffness of the muscles throughout the week was opposite to the general tendency to decrease in postcompetition situations and RF increased their muscle stiffness, significantly at the end of the week (Figure 3). This increase in stiffness can be justified by chronic muscle overload. This can occur due to sustained contractions over time and repetitive activity, usually characterized by strenuous physical activity required by certain sports disciplines, as in our case, when fatigue accumulates throughout a competitive week. This overload condition is involved in an increase in tension and stiffness in the myofascial tissue [65]. Although this increase is poorly documented in the scientific literature, some authors argue that during physical exercise, Ca^2+^ ions accumulate in the sarcoplasm of the muscle cell sarcoplasm, contributing to muscle contractures. Therefore, these contractures can be attenuated with a rest recovery period [32, 65].

### 4.1. Limitations

Our study has some limitations, such as a small sample size and the lack of objective markers of stress and fatigue levels during and after competition.

## 5. Conclusion

We found a decrease in muscle tone and stiffness at the end of the competition among professional female cyclists. These effects on muscle properties can be caused both by precompetition stress and by neuromuscular fatigue after long-term physical exercise. The VL followed by the RF are the muscles that obtained the most significant changes in biomechanical properties and were also the most activated muscles during cycling. All muscles analysed VL, VM, RF, BF, LG, and MG decreased their muscle stiffness after one competition. Generally, basal elasticity improved across the board immediately after the competition and continued to improve until the end of the week.

## Figures and Tables

**Table 1:** Anthropometric description and competition experience.

Variables	Mean ± SD (*n* = 6)	Min-Max
Age, years	25.0 ± 5.6	19.0–35.0
Weight, kg	57.7 ± 5.1	51.0–64.0
Height, cm	166.8 ± 4.0	159.0–170.0
BMI	20.7 ± 1,7	17.9–22.2
Competition experience, years	8.9 ± 9.8	1.5–28.0

BMI = body mass index, SD = standard deviation.

**Table 2:** Study design.

FP	R1	C1	R2	C2
Basal	Basal	Basal	Basal	Basal
Pre		Pre		Pre
Post		Post		Post

FP = familiarization phase, R1/R2 = rest day 1/rest day 2, C1/C2 = Competition 1/competition 2.

**Table 3:** HR, power output and km (*n* = 6).

Parameters	FP (108 km)	C1 (120,7 km)	C2 (126,7 km)
HR (bpm)	167	159	168
HR max (bpm)	194	193	192
Power output (W)	185	157	183
Power output max (W)	731	696	637

Heart rate = heart rate, bpm = beat per minute, W = watts, FP = familiarization phase, C1 = competition 1, C2 = competition 2.

**Table 4:** Muscle tone, stiffness, and elasticity of the 6 muscles before and after competition 1 and competition 2 (*n* = 6).

	C1		C2	
	PRE	POST	PRE	POST
*VL*				
Tone (Hz)	15.43 [13.57–16.45]	14.57 [12.84–15.07] ^*∗*^	14.78 ± 1.52	14.10 ± 1.14 ^*∗*^
Stiffness (N/m)	275.50 ± 22.13	255.90 ± 20.27 ^*∗*^	268.17 ± 36.44	256.13 ± 26.34
Elasticity (log D)	1.33 ± 0.18	1.18 ± 0.15 ^*∗*^	1.21 ± 0.17	1.20 ± 0.21

*VM*				
Tone (Hz)	13.61 ± 1.09	13.17 ± 1.68	13.25 ± 1.46	12.54 ± 1.38 ^*∗*^
Stiffness (N/m)	242.07 ± 20.29	224.90 ± 31.53	223.10 [191.40–274.40]	200.40 [144.60–261.20] ^*∗*^
Elasticity (log D)	1.24 ± 0.15	1.14 ± 0.18	1.17 [1.14–1.41]	1.05 [0.84–1.29] ^*∗*^

*RF*				
Tone (Hz)	14.02 ± 1.11	13.95 ± 1.58	14.01 ± 1.34	13.19 ± 0.65
Stiffness (N/m)	247.37 ± 25.72	232.00 ± 32.11	252.43 ± 29.50	225.53 ± 17.13 ^*∗*^
Elasticity (log D)	1.40 ± 0.25	1.21 ± 0.15 ^*∗*^	1.35 ± 0.18	1.18 ± 0.19 ^*∗*^

*BF*				
Tone (Hz)	14.85 ± 1.54	13.85 ± 1.14	14.64 ± 0.77	14.67 ± 1.53
Stiffness (N/m)	264.13 ± 34.74	236.97 ± 31.57 ^*∗*^	260.83 ± 24.25	256.03 ± 36.76
Elasticity (log D)	1.35 ± 0.18	1.26 ± 0.13	1.32 ± 0.16	1.25 ± 0.20

*LG*				
Tone (Hz)	14.52 ± 1.17	13.98 ± 0.61	14.83 ± 0.90	13.76 ± 0.62 ^*∗*^
Stiffness (N/m)	259.10 ± 21.33	239.00 ± 12.61 ^*∗*^	261.40 ± 38.20	237.53 ± 20.01
Elasticity (log D)	1.20 ± 0.18	1.19 ± 0.10	1.23 ± 0.16	1.19 ± 0.17

*MG*				
Tone (Hz)	16.26 ± 1.24	15.14 ± 1.12 ^*∗*^	15.84 ± 1.46	14.69 ± 0.94
Stiffness (N/m)	270.60 ± 15.88	245.50 ± 15.88 ^*∗*^	261.23 ± 29.47	244.23 ± 18.62
Elasticity (log D)	1.23 ± 0.06	1.23 ± 0.06	1.34 ± 0.27	1.28 ± 0.22

Data are expressed as mean ± SD except for cases where the Shapiro-Wilk test failed to demonstrate a normal distribution of the data, in such cases the median and ranges are presented  ^*∗*^*P* ≤ 0.05. VL = m. vastus lateralis, VM = m. vastus medialis, RF = m. rectus femoris, BF = m. biceps femoris, LG = m. lateral gastrocnemius, MG = m. medial gastrocnemius, C1 = competition 1, C2 = competition 2.

**Table 5:** Muscle tone, stiffness, and elasticity of the 6 muscles during 5 days of competition week (*n* = 6).

	FP	R1	C1	R2	C2
*VL*					
Tone (Hz)	14.84 ± 1.38	14.46 ± 0.80	14.86 ± 1.35	14.46 ± 1.11	15.06 ± 1.12
Stiffness (N/m)	272.97 ± 21.84	265.80 ± 15.19	275.13 ± 25.21	263.40 ± 27.76	272.47 ± 27.90
Elasticity (log D)	1.47 ± 0.20	1.40 ± 0.17	1.42 ± 0.24	1.34 ± 0.19	1.19 ± 0.20 ^*∗*^

*VM*					
Tone (Hz)	13.68 ± 1.36	13.34 ± 1.03	13.54 ± 1.17	13.42 ± 1.11	13.16 ± 0.91
Stiffness (N/m)	234.20 ± 25.23	239.90 ± 23.13	241.80 ± 22.22	235.27 ± 17.93	234.27 ± 20.06
Elasticity (log D)	1.35 ± 0.12	1.39 ± 0.20	1.38 ± 0.16	1.36 ± 0.15	1.29 ± 0.20

*RF*					
Tone (Hz)	14.07 ± 1.34	13.99 ± 1.11	13.86 ± 0.94	13.92 ± 1.09	14.12 ± 0.97
Stiffness (N/m)	242.97 ± 27.47	242.87 ± 24.42	247.27 ± 17.99	245.50 ± 14.84	262.13 ± 23.11 ^*∗*^
Elasticity (log D)	1.43 ± 0.18	1.42 ± 0.15	1.49 ± 0.17	1.44 ± 0.16	1.51 ± 0.26

*BF*					
Tone (Hz)	14.30 ± 0.98	14.18 ± 1.19	14.02 ± 0.88	14.17 ± 1.10	13.98 ± 0.90
Stiffness (N/m)	248.30 ± 22.16	249.77 ± 31.92	250.00 ± 22.20	246.03 ± 33.12	249.27 ± 28.23
Elasticity (log D)	1.37 ± 0.21	1.37 ± 0.15	1.31 ± 0.18	1.30 ± 0.20	1.33 ± 0.21

*LG*					
Tone (Hz)	14.08 ± 0.62	14.27 ± 1.08	14.79 ± 0.95	14.62 ± 0.95	14.36 ± 0.44
Stiffness (N/m)	246.37 ± 13.91	242.83 ± 14.21	249.40 ± 17.65	245.67 ± 17.67	245.87 ± 12.50
Elasticity (log D)	1.34 ± 0.16	1.21 ± 0.14	1.25 ± 0.16	1.26 ± 0.17	1.26 ± 0.19

*MG*					
Tone (Hz)	14.75 ± 0.82	15.70 ± 1.09	15.75 ± 0.93	15.66 ± 1.09	14.90 ± 0.88
Stiffness (N/m)	244.37 ± 14.40	248.40 ± 17.35	249.93 ± 13.03	245.27 ± 20.89	240.20 ± 16.28
Elasticity (log D)	1.47 ± 0.12	1.34 ± 0.12	1.38 ± 0.17	1.38 ± 0.20	1.35 ± 0.22

Values are mean ± SD,  ^*∗*^*P* ≤ 0.05 compared to day 1. VL = m. vastus lateralis, VM = m. vastus medialis, RF = m. rectus femoris, BF = m. biceps femoris, LG = m. lateral gastrocnemius, MG = m. medial gastrocnemius.

## Data Availability

The raw data were used to support the findings of this study and are included within the supplementary information files.

## Supplementary Materials

Supplementary Materials Supplementary file #1: muscle basal properties. Muscle tone, stiffness, and elasticity in the familiarisation phase. Supplementary file #2: muscle parameters before and after C1.pdf. Muscle tone, stiffness, and elasticity before and after Competition 1. Supplementary file #3: muscle parameters before and after C2.pdf. Muscle tone, stiffness, and elasticity before and after Competition 2.
