# Supplementary material for: Muscle Tone, Stiffness, and Elasticity in Elite Female Cyclists after Consecutive Short Competitions
Source: Transl Sports Med. 2024 Apr 2;2024:2434494. doi: 10.1155/2024/2434494 (PMC11023733; doi:10.1155/2024/2434494)
Supplement: Supplementary Materials — Supplementary file #1: muscle basal properties. Muscle tone, stiffness, and elasticity in the familiarisation phase. Supplementary file #2: muscle parameters before and after C1.pdf. Muscle tone, stiffness, and elasticity before and after Competition 1. Supplementary file #3: muscle parameters before and after C2.pdf. Muscle tone, stiffness, and elasticity before and after Competition 2. [file 2434494.f1.zip › Muscle Basal Parameters.pdf]

Subject	Muscle	Frequency 1	Stiffness 1	Decrement 1	Frequency 5	Stiffness 5	Decrement 5
1	Vastus lt	16.16	307.2	1.65	16.44	316.0	1.48
2	Vastus lt	16.44	272.0	1.44	14.98	276.6	1.15
3	Vastus lt	13.86	262.8	1.62	14.61	252.0	0.94
4	Vastus lt	14.52	283.8	1.54	14.73	262.2	1.26
5	Vastus lt	12.84	241.6	1.49	13.39	238.4	1.31
6	Vastus lt	15.24	270.4	1.10	16.20	289.6	1.00
1	Vastus m	16.06	281.6	1.54	14.77	263.8	1.56
2	Vastus m	12.62	224.6	1.36	13.54	244.0	1.49
3	Vastus m	12.34	210.6	1.25	12.11	206.6	1.06
4	Vastus m	14.34	239.8	1.36	12.79	227.4	1.15
5	Vastus m	13.57	218.8	1.37	12.88	221.4	1.29
6	Vastus m	13.16	229.8	1.20	12.89	242.4	1.16
1	Rect Femoris	16.32	268.8	1.75	16.02	302.4	1.82
2	Rect Femoris	14.59	266.2	1.48	14.06	273.4	1.70
3	Rect Femoris	14.43	260.6	1.29	14.05	260.0	1.20
4	Rect Femoris	12.79	205.8	1.27	13.49	245.4	1.47
5	Rect Femoris	12.92	213.4	1.45	13.43	238.6	1.67
6	Rect Femoris	13.35	243.0	1.33	13.65	253.0	1.21
1	Bic Fem c br	15.55	274.0	1.72	14.75	277.0	1.50
2	Bic Fem c br	14.92	265.4	1.43	15.10	284.4	1.62
3	Bic Fem c br	14.65	243.8	1.15	13.91	248.2	1.12
4	Bic Fem c br	13.14	212.2	1.21	13.33	213.8	1.11
5	Bic Fem c br	13.14	237.8	1.41	12.66	222.4	1.35
6	Bic Fem c br	14.39	256.6	1.28	14.15	249.8	1.27
1	Gastr c lt	13.86	242.4	1.33	14.20	237.2	1.33
2	Gastr c lt	14.28	255.4	1.33	14.68	243.0	1.07
3	Gastr c lt	15.06	257.6	1.12	15.08	263.4	1.03
4	Gastr c lt	13.90	235.2	1.36	14.29	229.4	1.21
5	Gastr c lt	13.18	226.4	1.28	13.90	245.4	1.42
6	Gastr c lt	14.18	261.2	1.60	14.03	256.8	1.48
1	Gastr c m	15.10	252.8	1.62	16.13	255.8	1.50
2	Gastr c m	14.74	240.6	1.37	13.43	214.4	1.05
3	Gastr c m	15.54	245.2	1.33	15.22	254.4	1.18
4	Gastr c m	14.64	237.8	1.38	14.95	231.0	1.28
5	Gastr c m	13.23	223.6	1.49	15.02	235.2	1.46
6	Gastr c m	15.25	266.2	1.60	14.65	250.4	1.62
