# Supplementary material for: Muscle Tone, Stiffness, and Elasticity in Elite Female Cyclists after Consecutive Short Competitions
Source: Transl Sports Med. 2024 Apr 2;2024:2434494. doi: 10.1155/2024/2434494 (PMC11023733; doi:10.1155/2024/2434494)
Supplement: Supplementary Materials — Supplementary file #1: muscle basal properties. Muscle tone, stiffness, and elasticity in the familiarisation phase. Supplementary file #2: muscle parameters before and after C1.pdf. Muscle tone, stiffness, and elasticity before and after Competition 1. Supplementary file #3: muscle parameters before and after C2.pdf. Muscle tone, stiffness, and elasticity before and after Competition 2. [file 2434494.f1.zip › Muscle parameters before-after C1.pdf]

Subject	Muscle	Frequency PRE	Frequency POST	Stiff PRE	Stiff POST	Decrem PRE	Decrem POST
1	Vast lateral	16.16	15.07	297.2	281.4	1.65	1.28
2	Vast lateral	15.35	14.66	273.8	257.2	1.37	1.24
3	Vast lateral	14.07	13.30	267.6	238.2	1.14	1.00
4	Vast lateral	15.50	14.78	275.8	269.6	1.21	1.12
5	Vast lateral	13.57	12.84	239.0	226.6	1.40	1.38
6	Vast lateral	16.45	14.47	299.6	262.4	1.22	1.03
1	Vast medial	15.73	16.55	280.0	285.8	1.47	1.46
2	Vast medial	12.84	12.81	224.0	224.0	1.32	1.10
3	Vast medial	12.88	12.18	226.2	208.8	1.25	1.14
4	Vast medial	13.60	12.58	245.4	212.2	1.01	1.03
5	Vast medial	13.57	12.75	237.2	222.6	1.20	1.15
6	Vast medial	13.04	12.15	239.6	196.0	1.21	0.94
1	Recte femoral	15.40	16.72	277.4	275.4	1.84	1.44
2	Recte femoral	14.56	14.34	254.2	248.4	1.47	1.24
3	Recte femoral	14.79	14.3	263.0	255.4	1.33	1.18
4	Recte femoral	12.34	12.88	201.8	201.2	1.21	1.00
5	Recte femoral	13.73	13.11	241.2	214.0	1.41	1.27
6	Recte femoral	13.31	12.32	246.6	197.6	1.13	1.12
1	Biceps femoral	15.30	14.94	285.4	268.8	1.47	1.42
2	Biceps femoral	14.68	14.89	259.8	260.0	1.36	1.35
3	Biceps femoral	15.17	14.18	277.2	249.8	1.31	1.22
4	Biceps femoral	14.23	13.02	235.8	195.0	1.03	1.06
5	Biceps femoral	12.50	12.00	215.4	199.8	1.40	1.30
6	Biceps femoral	17.23	14.09	311.2	248.4	1.53	1.21
1	Gastrc lateral	14.80	14.00	258.4	237.6	1.10	1.13
2	Gastrc lateral	13.73	14.50	234.6	235.6	1.24	1.21
3	Gastrc lateral	16.56	14.86	291.4	261.2	0.93	1.10
4	Gastrc lateral	14.57	13.77	258.0	233.4	1.15	1.13
5	Gastrc lateral	13.14	13.28	238.6	223.4	1.30	1.18
6	Gastrc lateral	14.30	13.47	273.6	242.8	1.47	1.38
1	Gastrc medial	16.90	16.34	309.8	268.2	1.25	1.27
2	Gastrc medial	15.44	15.52	234.4	244.0	1.15	1.20
3	Gastrc medial	18.23	15.88	297.0	254.2	1.08	1.15
4	Gastrc medial	15.65	14.83	261.0	238.2	1.01	1.25
5	Gastrc medial	14.75	13.13	239.2	220.8	1.32	1.20
6	Gastrc medial	16.60	15.12	282.2	247.6	1.49	1.33
